# Elevated expression of ciRS-7 in peripheral blood mononuclear cells from rheumatoid arthritis patients

**DOI:** 10.1186/s13000-019-0783-7

**Published:** 2019-02-02

**Authors:** Xinyi Tang, Jiemin Wang, Xin Xia, Jie Tian, Ke Rui, Huaxi Xu, Shengjun Wang

**Affiliations:** 1 Department of Laboratory Medicine, The Affiliated People’s Hospital, Jiangsu University, Zhenjiang, 212002 China; 2 Institute of Laboratory Medicine, Jiangsu Key Laboratory for Laboratory Medicine, Jiangsu University School of Medicine, Zhenjiang, 212013 Jiangsu Province China

**Keywords:** ciRS-7, miR-7, mTOR, Rheumatoid arthritis

## Abstract

**Background:**

Circular RNAs (circRNAs) represent a class of widespread and variety endogenous RNAs that may regulate gene expression. Thousands of mammalian circRNAs harbor miRNA response elements (MREs), suggesting a potential role as competitive endogenous RNAs (ceRNAs). Recent studies have demonstrated that ciRS-7 (circular CDR1 antisense), which acts as a powerful miR-7 sponge, contains more than 70 putative binding sites for miR-7 and may inhibit its target genes. The aim of this preliminary study was to investigate the expression of ciRS-7 in patients with rheumatoid arthritis (RA) as well as the correlation between ciRS-7 and the target genes of miR-7.

**Methods:**

Eighteen patients with RA and 14 healthy controls were enrolled in the current study. The relative expression of ciRS-7, miR-7, miR-671 and mTOR in peripheral blood mononuclear cells (PBMCs) from these samples were detected by real-time PCR.

**Results:**

We found that ciRS-7 was significantly increased in RA patients and could potentially differentiate the RA patients from healthy controls. Additionally, the expression of mTOR, one of the miR-7 target genes, had positive and negative relationships with ciRS-7 and miR-7 expression, respectively. Notably, the relative expression of miR-671, which mediated the regulation of circular CDR1 antisense homeostasis, was significantly decreased in RA patients.

**Conlusion:**

Downregulated miR-671 may influence the level of ciRS-7 in RA patients. Enhanced ciRS-7 could inhibit the function of miR-7 and further relieve the inhibitory effect of miR-7 on mTOR.

## Background

Circular RNAs (circRNAs) are RNA molecules and important regulators of miRNA activity, which are formed by back-splice events and thus present as covalently closed continuous loops. MiRNAs are short, single stranded, non-coding RNAs involved in the post-transcriptional regulation of gene expression. ciRS-7 is one of the most widely studied circular RNAs [1, 2]. It has an antisense orientation with respect to the CDR1 gene, cerebellum degeneration-related antigen 1 (CDR34), which has been implicated in autoimmune neurologic disorder since the late 1980s [3, 4]. Notably, ciRS-7 harbors more than 70 conventional miR-7 binding sites, acts as a designated miR-7 inhibitor/sponge, and efficiently reduces miR-7 activity, consistent with the competing eRNA (ceRNA) hypothesis [5]. However, in contrast to classical ceRNAs, ciRS-7 has no accessible termini and could be resistant to miRNA-mediated RNA destabilization [6]. This observation suggests that ciRS-7 might participate in various miR-7-dependent pathways in autoimmune diseases.

Rheumatoid arthritis (RA) is a chronic, inflammatory, autoimmune disorder characterized by synovial inflammation and adjacent cartilage and bone destruction that may result in severe lifelong disability [7, 8]_ENREF_7. RA is driven by dysregulated innate and adaptive immune responses that provide an increasingly rich therapeutic resource. PI3K/Akt/mTOR is one of the most important intracellular signaling pathways in mammalian cells [9, 10]. The role of the PI3K/AKT/mTOR pathway in promoting aggressive immune cell and synoviocyte proliferation and altered innate immunity in inflammatory arthritis was recently reviewed. [11–16] Importantly, a correlation was found between activated mTOR signaling and the number of osteoclasts in RA patients [17]. In addition, one miR-7 target site was found in the mTOR 3’UTR, and miR-7 was reported to efficiently regulate the PI3K/Akt pathway by targeting PIK3CD, mTOR and p70S6K [18, 19] .

In this preliminary study, we aimed to detect the variation of ciRS-7 in RA patients and analyze the correlation between mTOR and ciRS-7 in peripheral blood mononuclear cells (PBMCs). Using this approach, we hoped to identify the potential diagnostic value of ciRS-7 in RA patients.

## Material and method

### Patients and healthy controls

A total of 18 patients with RA were enrolled in the study. The major clinical data of these patients are shown in Table 1. All patients met the American College of Rheumatology (ACR) 1987 and The European League against Rheumatism (EULAR) 2009 revised criteria for the classification of RA and without any treatment. All blood samples were collected under fasting conditions in the morning. The concentrations of anti-CCP antibody in the serum from RA patients were determined by ELISA (ORMOND, Germany) using an ELISA reader (Bio-Rad). Fourteen healthy control subjects were free of chronic pain, cardiovascular complaints, or other chronic inflammatory diseases. PBMCs were prepared from all patients and healthy controls for subsequent measurement.

**Table 1:** Clinical features of RA patients included in this study

	RA	Range
n	18	
Gender(F/M)	12/6	
Age	52.56 ± 17.32	31–84
anti-CCP(IU/ml)	26,840 ± 34,351	1444–111,563

Data correspond to the arithmetic mean ± SD, M: Male; F: Female

All samples were collected in accordance with the regulations and approval of the Affiliated People’s Hospital of Jiangsu University.

### RNA extraction and qRT-PCR

PBMCs were isolated by density-gradient centrifugation over Ficoll-Hypaque solution [20]. TRIzol reagent (Invitrogen, Carlsbad, CA) was added to isolate PBMCs. Total RNA isolation was performed according to the manufacturer’s instruction. Reverse transcription (Toyobo, Osaka, Japan) was performed according to the manufacturer’s instructions for synthesizing cDNA [21]. The primers in the reverse transcription kit (Toyobo, Osaka, Japan) consisted of oligo-dTN and random primers. The cDNA obtained by priming with random primers was used for ciRS-7 detection, and the cDNA obtained by priming with oligo-dTN was used for mTOR mRNA detection. The miRNA qRT-PCR Primer Set (RiboBio, Guangzhou, China) and M-MLV Reverse Transcriptase (TaKaRa, Dalian, China) were used for miR-7 and miR-671 reverse transcription.

qRT-PCR was performed in duplicate using the Bio-Rad SYBR Green Super mix (Bio-Rad, Hercules, CA). Primer sequences were as follows: mTOR, sense, 5’-CGCTGTCATCCCTTTATCG-3′; antisense, 5’-ATGCTCAAACACCTCCACC-3′, ciRS-7, sense, 5’-CCTGGGCTCCTCGCCTGACC-3′; antisense, 5′- TCTCTCTGCCCTCAGCCTTGCC-3′, GAPDH, sense, 5’-CAA GTC CCG CCG CTC CAT TAC CAA-3′; anti-sense, 5’-CCA CAG CCG TCC CAG TCA CAG TGG-3′. The expression levels of ciRS-7 and mTOR mRNA were normalized to the endogenous expression of GAPDH. The expression levels of miR-7 and miR-671 were normalized to the expression of U6. Data were analyzed by Bio-Rad CFX Manager software.

### Statistical analyses

Student’s unpaired or paired t test was performed to determine whether there was a statistically significant difference between the RA group and healthy group. Correlations between variables were determined by Spearman’s correlation coefficient. Receiver-operator characteristic (ROC) curve analysis was used to assess the diagnostic value, including AUC, sensitivity, and specificity. *P* < 0.05 was considered statistically significant. All statistical analyses were performed with GraphPad Prism5 software (GraphPad Software, Inc., San Diego, CA).

## Results

### Increased expression of ciRS-7 in patients with RA

**Fig. 1:**
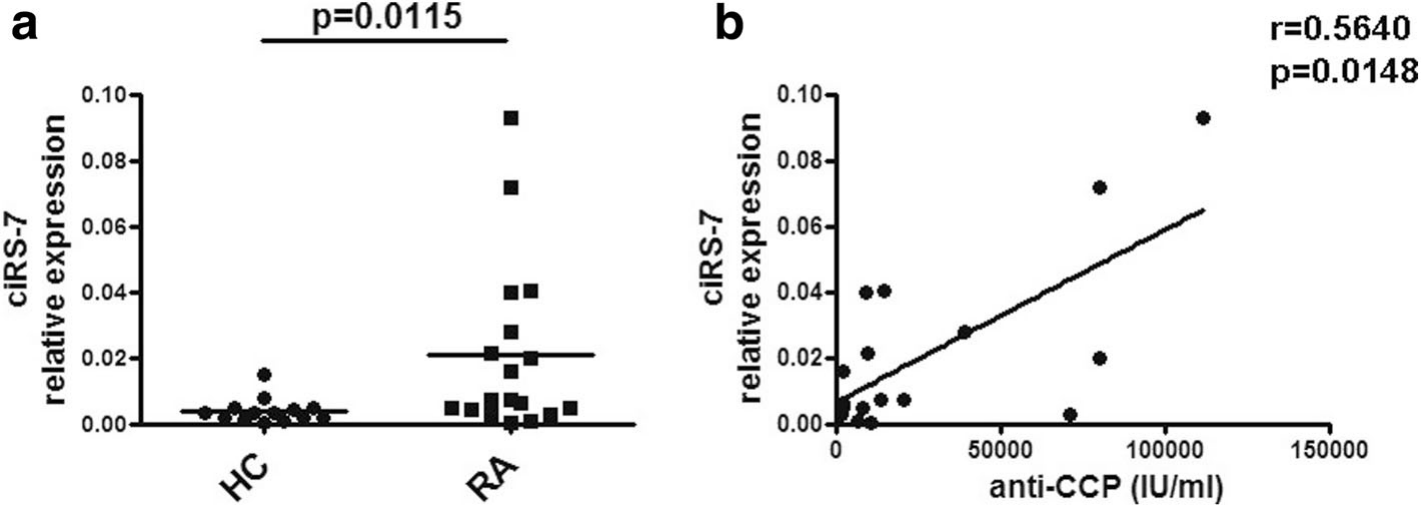
Increased expression of ciRS-7 in patients with RA. **a** The relative expression of ciRS-7 in PBMCs from RA patients and controls was detected by qRT-PCR. **b** The correlation between the relative expression of ciRS-7 in PBMCs and concentration of anti-CCP in the serum from RA patients

To study ciRS-7 expression in RA patients, peripheral blood was obtained from RA patients. As shown in Fig. 1a, the relative expression of ciRS-7 was significantly higher in patients than healthy controls. Increased anti-CCP antibody is a generally accepted objective clinical manifestation of RA patients. Numerous clinical studies have shown that anti-CCP antibody is an index of RA prognosis that is extremely specific for RA diagnosis. We analyzed the correlation of ciRS-7 expression with anti-CCP antibody concentration in RA patients and found a positive correlation between relative expression of ciRS-7 and plasma levels of anti-CCP antibody in RA patients (Fig. 1b).

### The comparison of miR-7 expression between RA patients and control individuals

**Fig. 2:**
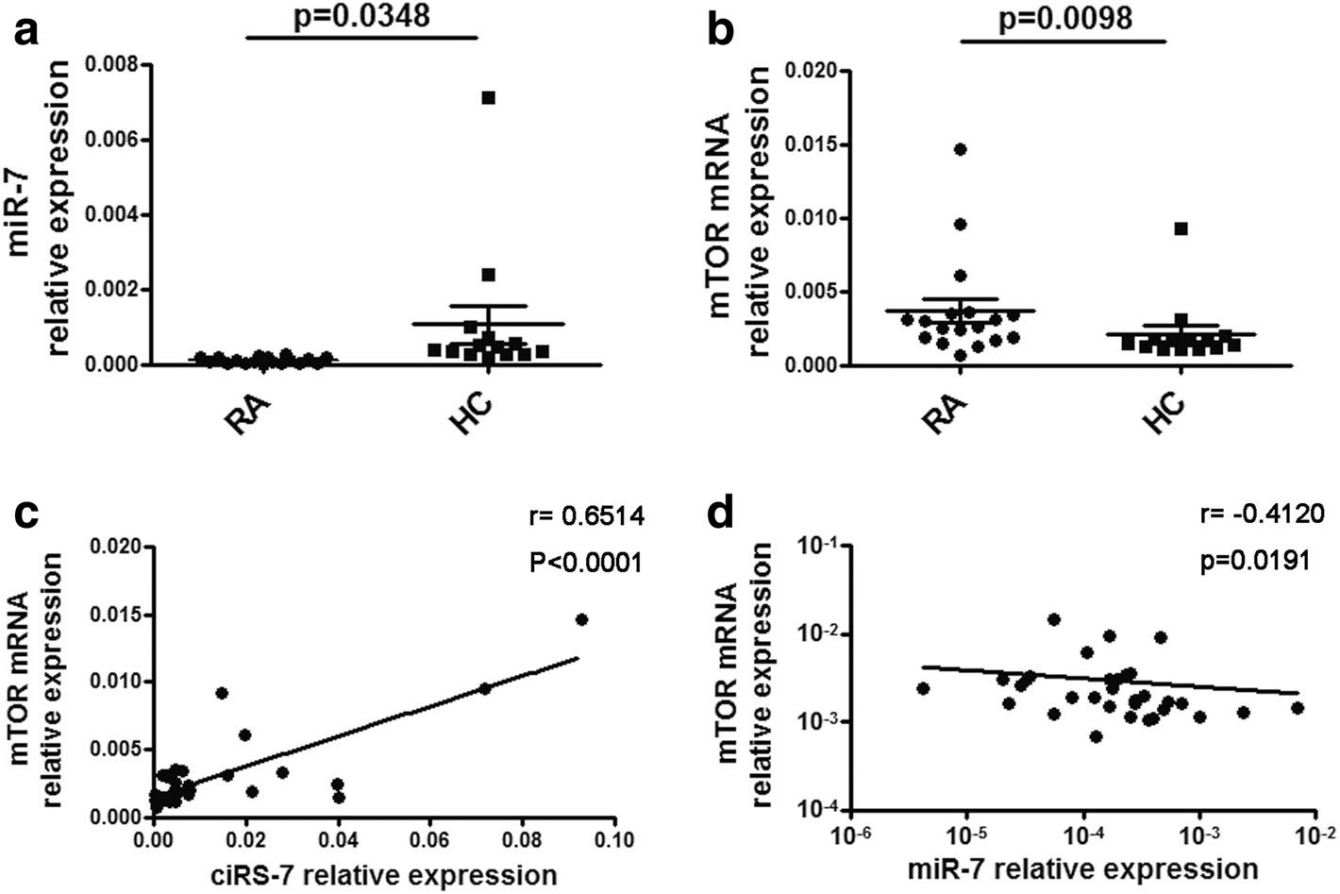
The comparison of miR-7 expression between RA patients and control individuals. **a** The miR-7 levels in PBMCs from RA patients and controls were detected by qRT-PCR. **b** The relative expression of mTOR in PBMCs from RA patients and healthy controls was detected by qRT-PCR. **c** The correlation between the relative expression of ciRS-7 and mTOR mRNA in PBMCs. **d** The correlation between the relative expression of miR-7 and mTOR mRNA in PBMCs

Increased ciRS-7 resulted in reduced miR-7 activity and increased levels of miR-7-targeted transcripts. The expression of ciRS-7 was increased in RA patients as described above, and we decided to examine the expression of ciRS-7. As shown in Fig. 2a, the relative expression of miR-7 significantly decreased in RA patients. miR-7a was reported to target the 3’-UTR of several components of the mTOR molecule and may modulate their expression and function. In this study, the relative expression of mTOR mRNA was detected in PBMCs from RA patients. The mTOR expression is higher in the PBMCs from RA patients compared with healthy controls (Fig. 2b). Notably, we found that expression of mTOR in PBMCs from RA patients and healthy controls had a significant positive correlation with ciRS-7 expression and a negative correlation with miR-7 expression (Fig. 2c and d). Based on these results, we hypothesized that increased expression of ciRS-7 in RA peripheral blood may act as a sponge that could absorb a certain amount of miR-7 and further upregulate mTOR mRNA expression.

### Downregulation of miR-671 in RA patients

**Fig. 3:**
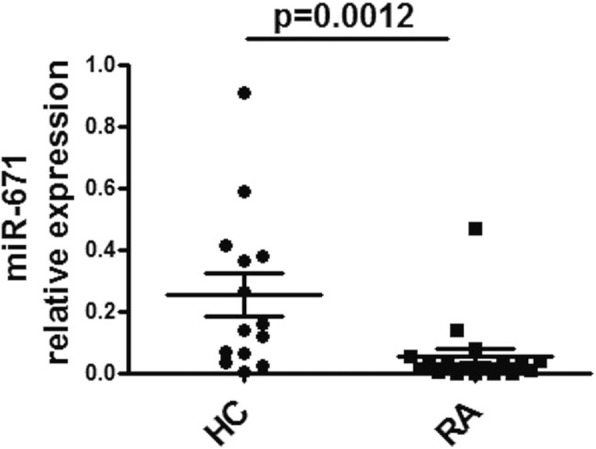
Downregulation of miR-671 in RA patients. The expression of miR-671 in PBMCs from RA patients and healthy controls was detected by qRT-PCR

CDR1 was identified as a putative target gene of miR-671. The target site is highly complementary to mature miR-671 and is situated in the antisense direction proximal to the CDR1 transcription start site [22]. As determined by northern blot analysis, ectopically expressed CDR1 antisense transcripts were almost completely eliminated by miR-671. Based on these results, the relative expression of miR-671 was detected in PBMCs from RA patients to explore the mechanisms of ciRS-7 enhancement. As shown in Fig. 3, the relative expression of miR-671 was significantly reduced in patients with RA.

### Evaluation of the diagnostic value of ciRS-7 for RA

**Fig. 4:**
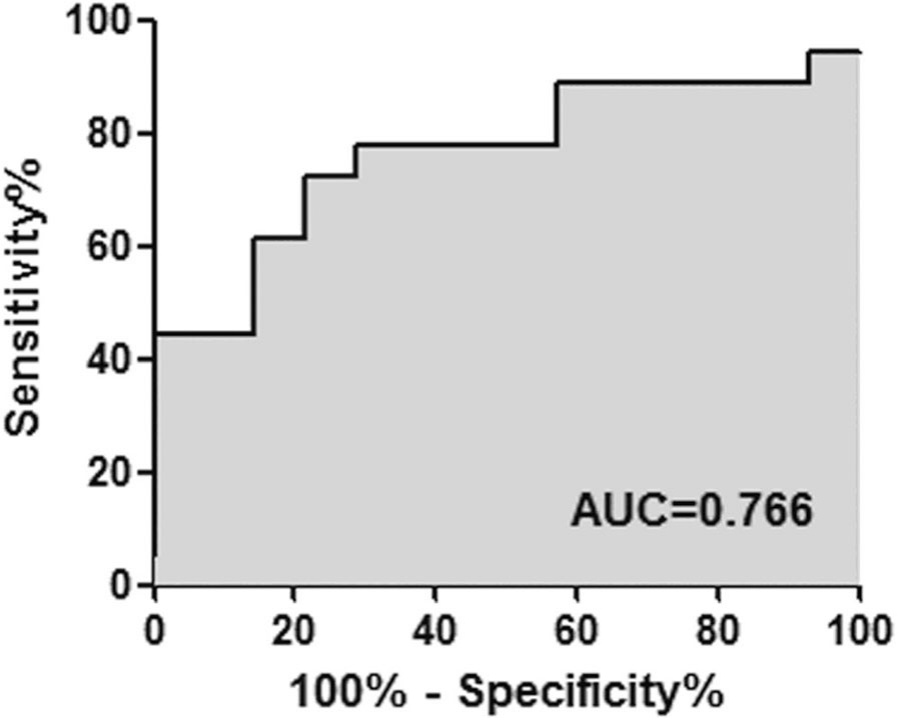
ROC curve analysis using ciRS-7 to distinguish RA patients. ROC curve analysis was performed to show the AUC of ciRS-7

To assess the efficiency of ciRS-7 as a diagnostic marker for RA detection, we performed ROC curve analysis on ciRS-7. Figure 4 shows that the ROC curves of ciRS-7 could distinguish RA patients from healthy controls. For this, the sensitivity was 77.8% and the specificity was 78.6% with an AUC of 0.766.

These data indicated that the ciRS-7, which showed the ability to differentiate RA patients from healthy controls, could act as a suitable biomarker for RA diagnosis.

## Discussion

Several studies have elucidated the relationship between circRNAs and cancers [23, 24]. Cir-ITCH enhanced the expression of ITCH and further inhibited the Wnt/β-catenin pathway. Interference of cir-ITCH may promote development of esophageal squamous cell carcinoma (ESCC) and colorectal cancer (CRC) through defective Wnt signaling [25, 26]. Because of their stability, circRNAs are potential biomarkers for diagnosis of diseases. Thus far, no studies have examined the relationship between circRNAs and autoimmune diseases. Several researchers have shown that various microRNAs are involved RA [27–29]. Because these molecules can function as ceRNAs, alteration of circRNA expression may affect the function of related miRNAs and then alter the target genes of this miRNA. We hypothesized that changes in circRNAs may be involved the progression of RA through modification of miRNAs.

Interestingly, the results showed relative expression of ciRS-7 was significantly higher in peripheral blood from RA patients. Thus, we investigated the factors that caused this increase in ciRS-7 in patients with RA. A relevant article reported that inhibiting miR-671 by anti-mir-671 could activate CDR1 in HEK293 cells. Additionally, the researchers confirmed that CDR1NAS is cleaved and degraded in a miR-671-dependent manner [22]. Their data demonstrated that sequence-specific miR-671 mediated the regulation of circular CDR1 antisense homeostasis.

On the basis of these results, we detected the relative expression of miR-671 in the PBMCs from RA patients and compared them with healthy controls. Interestingly, the relative expression of miR-671 was significantly decreased in RA patients. Combined with the results of previous articles, we hypothesized that insufficient miR-671 in RA patients may be one factor that causes the high expression of ciRS-7.

To better elucidate the potential impact of enhanced ciRS-7 levels in the peripheral blood of patients with RA, the expression of miR-7, which could be intensely absorbed by ciRS-7, was also detected. As expected, the relative expression of miR-7 in PBMCs from RA patients was significantly decreased compared with healthy controls. These results suggest that the miR-7 decrease was caused by elevated ciRS-7. However,no studies have shown that miR-7 that was absorbed by ciRS-7 could not be detected. Thus, determining the influence of ciRS-7 on the function of miR-7 and the expression of its target genes is more acceptable. The abnormal proliferation of synovial fibroblasts in RA patients was reported to be PI3K/mTOR signaling-dependent. We investigated whether aberrant mTOR, one of the target genes of miR-7, was observed. Therefore, the relative expression of mTOR was detected. Consistent with our expectations, mTOR expression significantly increased in RA patients. Moreover, it had positive and negative relationship with ciRS-7 and miR-7 expression, respectively, indicating that ciRS-7, by indirectly influencing expression of mTOR in peripheral blood, may be involved in RA. However, the detailed mechanism remains unclear, and whether ciRS-7 destroys the synovial balance or is passively affected by other potential risk factors requires further investigation. Moreover, the direct evidence on the impact of ciRS-7 on the function of miR-7 is still unclear. We hypothesized that ciRS-7 may inhibit the function of miR-7. In addition, we assessed the efficiency of ciRS-7 as a diagnostic marker for RA detection, and the results indicated that the ciRS-7 level could potentially differentiate the RA patients from controls.

This study only reveals an interesting phenomenon and provides clues for further research on the diagnostic value of circular RNA in RA. However, there are still limitations of this study. In addition to comparing the relative expression of ciRS-7 in PBMCs from RA patients and healthy controls, the ciRS-7 expression should be compared in RA patients with or without treatment to study the value of ciRS-7 in disease treatment evaluation. Generally, downregulated miR-671 may influence the level of ciRS-7 in RA patients, and enhanced ciRS-7 likely inhibits the function of miR-7 and further relieved the inhibitory effect of miR-7 on mTOR.

## Conclusions

To summarize, the level of ciRS-7 in RA patients may be influenced by lower expression of miR-671. Thereafter, enhanced ciRS-7 may inhibit the function of miR-7, and then further reduce the inhibitory effect of miR-7 on mTOR.

## Abbreviations

ceRNAs: Competitive endogenous RNAs
circRNAs: Circular RNAs
mTOR: Mammalian target of rapamycin
PBMCs: Peripheral blood mononuclear cells
qRT-PCR: Quantitative real time polymerase chain reaction
RA: Rheumatoid arthritis
